# Analyzing Short-Term Noise Dependencies of Spike-Counts in Macaque Prefrontal Cortex Using Copulas and the Flashlight Transformation

**DOI:** 10.1371/journal.pcbi.1000577

**Published:** 2009-11-26

**Authors:** Arno Onken, Steffen Grünewälder, Matthias H. J. Munk, Klaus Obermayer

**Affiliations:** 1 School of Electrical Engineering and Computer Science, Technische Universität Berlin, Berlin, Germany; 2 Bernstein Center for Computational Neuroscience Berlin, Berlin, Germany; 3 Centre for Computational Statistics and Machine Learning, University College London, London, United Kingdom; 4 Max Planck Institute for Biological Cybernetics, Tübingen, Germany; University of Freiburg, Germany

## Abstract

Simultaneous spike-counts of neural populations are typically modeled by a Gaussian distribution. On short time scales, however, this distribution is too restrictive to describe and analyze multivariate distributions of discrete spike-counts. We present an alternative that is based on copulas and can account for arbitrary marginal distributions, including Poisson and negative binomial distributions as well as second and higher-order interactions. We describe maximum likelihood-based procedures for fitting copula-based models to spike-count data, and we derive a so-called flashlight transformation which makes it possible to move the tail dependence of an arbitrary copula into an arbitrary orthant of the multivariate probability distribution. Mixtures of copulas that combine different dependence structures and thereby model different driving processes simultaneously are also introduced. First, we apply copula-based models to populations of integrate-and-fire neurons receiving partially correlated input and show that the best fitting copulas provide information about the functional connectivity of coupled neurons which can be extracted using the flashlight transformation. We then apply the new method to data which were recorded from macaque prefrontal cortex using a multi-tetrode array. We find that copula-based distributions with negative binomial marginals provide an appropriate stochastic model for the multivariate spike-count distributions rather than the multivariate Poisson latent variables distribution and the often used multivariate normal distribution. The dependence structure of these distributions provides evidence for common inhibitory input to all recorded stimulus encoding neurons. Finally, we show that copula-based models can be successfully used to evaluate neural codes, e.g., to characterize stimulus-dependent spike-count distributions with information measures. This demonstrates that copula-based models are not only a versatile class of models for multivariate distributions of spike-counts, but that those models can be exploited to understand functional dependencies.

## Author Summary

The brain has an enormous number of neurons that do not work alone but in an ensemble. Yet, mostly individual neurons were measured in the past and therefore models were restricted to independent neurons. With the advent of new multi-electrode techniques, however, it becomes possible to measure a great number of neurons simultaneously. As a result, models of how populations of neurons co-vary are becoming increasingly important. Here, we describe such a framework based on so-called copulas. Copulas allow to separate the neural variation structure of the population from the variability of the individual neurons. Contrary to standard models, versatile dependence structures can be described using this approach. We explore what additional information is provided by the detailed dependence. For simulated neurons, we show that the variation structure of the population allows inference of the underlying connectivity structure of the neurons. The power of the approach is demonstrated on a memory experiment in macaque monkey. We show that our framework describes the measurements better than the standard models and identify possible network connections of the measured neurons.

## Introduction

So far, it is still unknown which statistics are crucial for analysis in order to understand the neural code. One approach is to analyze simultaneous spike-counts of neural populations. Responses from populations of sensory neurons vary even when the same stimulus is presented repeatedly, and the variations between the simultaneous spike-counts are usually correlated (*noise correlations*) at least for neighboring neurons. These noise correlations have been subject of a substantial number of studies (see [1] for a review). For computational reasons, however, these studies typically assume Gaussian noise. Thus, correlated spike rates are generally modeled by multivariate normal distributions with a specific covariance matrix that describes all pairwise linear correlations.

**Figure 1:**
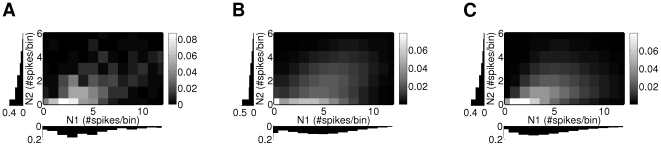
Modeling a spike-count distribution. (A) Normalized empirical distributions of spike-counts from a pair of neurons recorded in macaque prefrontal cortex (see Section “Materials and Methods”). The bin size was 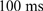. Gray values of the squares denote the number of occurrences of pairs of spike-counts (dark to bright corresponding to low to high, see scale bar). The corresponding marginals are plotted below and left of the coordinate axes. The distribution is based on 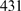 occurrences. (B) Joint distribution and marginals of the discretized and rectified multivariate normal distribution with the mean and covariance matrix set to the sample mean and sample covariance matrix. (C) Joint distribution and marginals of the best fitting Clayton copula (see Section “Multivariate Spike-Count Distributions Based on Copulas”, parameter: 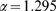) and negative binomial marginals (parameters: 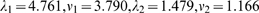).

For long time intervals or high firing rates, the average number of spikes is sufficiently large for the *central limit theorem* to apply and the normal distribution is a good approximation for the spike-count distributions. Several experimental findings, however, suggest that processing of sensory information can take place on shorter time scales, involving only tens to hundreds of milliseconds [2],[3]. In this regime the normal distribution is no longer a valid approximation:

1. Its marginals are continuous with a symmetric shape, whereas empirical distributions of real spike-counts tend to have a positive skew (see Figure 1A).
2. The normal distribution has to be heuristically modified in order to avoid positive probabilities for negative values which are not meaningful for spike-counts. This is a major issue for low rates for which the probability of negative values would be high.
3. The dependence structure of a multivariate normal distribution is always elliptical, whereas spike-count data often show a so-called tail-dependence with probability mass concentrated on one of the corners (see Figure 1A).
4. The multivariate normal distribution assumes second order correlations only. Although it was shown that pairwise interactions are sufficient for describing the spike-count distributions of retinal ganglion cells and cortex cells *in vitro* [4], there is evidence for significant higher order interactions of spike-counts recorded from cortical areas *in vivo* [5].

Though not widespread for modeling spike-counts, alternative models have been proposed in previous studies that have Poisson distributed marginals and separate parameters for higher order correlations, e.g. the multiple interaction process model [6] and the compound Poisson model [7]. Both models are point processes. In terms of their induced spike-count distributions these models are special cases of the multivariate Poisson latent variables distribution first introduced by Kawamura [8] and presented in a compact matrix notation by Karlis and Meligkotsidou [9]. Similar to the multivariate normal distribution this model has also a couple of shortcomings for spike-count modeling: (1) Only Poisson-marginals can be modeled. (2) Negative correlations cannot be represented. (3) The dependence structure is inflexible: features like tail dependence cannot be modeled.

We use and extend a versatile class of models for multivariate discrete distributions that overcome the shortcomings of the afore-mentioned models [10],[11]. These models are based on the concept of *copulas* [12], which allow to combine arbitrary marginal distributions using a rich set of dependence structures. In neuroscience they were also applied to model the distribution of continuous first-spike-latencies [13].

Figure 1 illustrates the copula concept using spike-count data from two real neurons. Figure 1A shows the bivariate empirical distribution and its two marginals. The distribution of the counts depends on the length of the time bin that is used to count the spikes, here 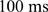. In the case considered, the correlation at low counts is higher than at high counts. This is called *lower tail dependence* [12]. Figure 1B shows the discretized and rectified multivariate normal distribution. On the other hand, the spike-count probabilities for a copula-based distribution (Figure 1C) correspond well to the empirical distribution in Figure 1A.

The paper is organized as follows. The next Section “Materials and Methods” contains a description of methodological details regarding the multivariate normal distribution, the multivariate Poisson latent variables distribution, the copula approach for spike-counts and the model fitting procedures. In this section we will also introduce a novel transformation for copula families. The method is innovative and yields a novel result. We will then describe the computational model used to generate synthetic data and the experimental recording and analysis procedures. In the Section “Results” copula-based models will be applied to artificial data generated by integrate-and-fire models of coupled neural populations and to data recorded from macaque prefrontal cortex (PFC) during a visual memory task. The appropriateness of the models is also investigated. The paper concludes with a discussion of the strengths and weaknesses of the copula approach for spike-counts.

## Materials and Methods

### Ethics Statement

All procedures were approved by the local authorities (Regierungspräsidium) and are in full compliance with the guidelines of the European Community (EUVD 86/609/EEC) for the care and use of laboratory animals.

### The Discretized Multivariate Normal Distribution

The multivariate normal (MVN) distribution is characterized by a probability density over continuous variables 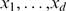 and its cumulative distribution function (CDF) with mean 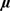 and covariance matrix 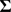 is given by
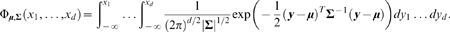



In order to apply it to spike-count distributions (which are discrete and non-negative) it is discretized and rectified (probability for negative values is set to zero). Its CDF is given by
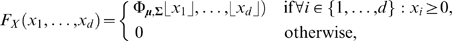
where 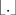 denotes the floor operation for the discretization. The probability mass function will have peaks at the zero count rows, due to the rectification of the CDF. It would be desirable to distribute the cut off mass equally to the complete domain. However, this is infeasible for more than three dimensions, because the necessary normalization term is computationally too time-consuming. Note that 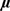 is no longer the mean of the distribution corresponding to 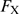, because the mean is shifted to larger values as 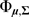 is rectified. This shift grows with the dimension 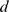.

### The Poisson Latent Variables Distribution

The Poisson latent variables distribution is characterized by a probability mass function (PMF) over non-negative integer variables 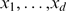 [9]. A random variable 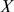 with this distribution is composed of 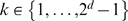 latent variables 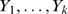. These latent variables are independent univariate Poisson distributed with rates 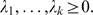 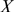 takes the form 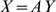, where 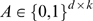 is a mixture matrix. The PMF of 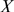 is then given by
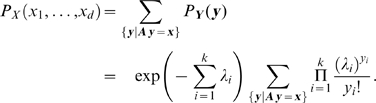



When we set 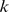 to 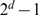 we can vary all pairwise and higher order interactions separately using the rates of the latent variables. However, only non-negative correlations can be modeled, because the rates of the latent variables are non-negative. Furthermore, the 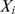 are marginally Poisson distributed.

### Copula Models of Multivariate Distributions

A copula is a cumulative distribution function (CDF) which is defined on the unit hypercube and which has uniform marginals [12]. Formally, a copula 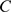 is defined as follows:

#### Definition 1

*A d*-copula *is a function* 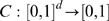 *such that* 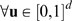 *:*

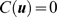
 *if at least one coordinate of* 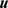 *is* 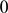.

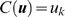
 *if all coordinates of* 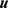 *are* 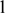 *except* 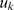.
3. *Let* 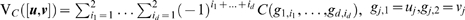 *, then* 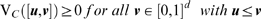 *.*

Property 3 states that the mass in every hypercube is non-negative. Together with property 1 it guarantees that 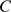 is a proper CDF on the unit hypercube, whereas property 2 ensures uniform marginals.

Copulas can now be used to couple arbitrary marginal CDFs to form a joint CDF. This is formalized in Sklar's Theorem [12],[14], which states:

#### Theorem 1

*Let* 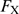 *be a d-dimensional cumulative distribution function with marginals* 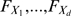 *. Then there exists a d-copula* 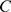 *such that for all* 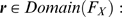


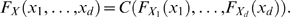




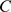
 *is unique, if* 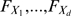 *are all continuous, and unique on* 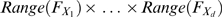, if 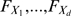 *are discrete.*

*Conversely, if* 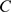 *is a d-copula and* 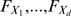 *are CDFs, then the function* 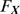 *defined by* 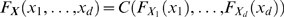 *is a d-dimensional CDF with marginals* 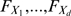.

Theorem 1 provides a way to construct multivariate distributions by attaching marginal CDFs to copulas. Copulas make an attachment possible, because they have continuous uniform marginals. In the univariate case a continuous uniform distribution on the unit interval can be easily transformed into any other distribution by applying the inverse of its CDF (inversion method). In the case of discrete marginal distributions, however, typical measures of dependence, such as Pearson's correlation coefficient or Kendall's 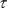 are effected by the shape of these marginals. This is due to the restricted uniqueness of the copula to the range of the discrete marginal distributions [15]. Moreover, an interpretation of the dependence structure for varying discrete marginals is difficult [15]. In this study, copula families are compared with respect to fixed marginals.

### Multivariate Spike-Count Distributions Based on Copulas

Our goal is to construct multivariate distributions for simultaneously recorded spike-counts that can model a wide range of dependence structures. Copulas make it possible to model multivariate distributions based on two distinct parts: the distributions of the individual elements and the dependence structure. Let us now assume that 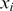 represents the spike-count of neuron 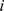 within a given interval. According to Theorem 1 we can then describe the joint cumulative distribution function of the spike counts 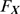 by choosing a copula 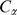 from a particular family, and by setting 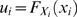 and 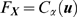. 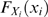 are the models of the marginal distributions, i.e. the cumulative distributions of spike-counts of the individual neurons. Often, the Poisson distribution is a good approximation to spike-count variations of single neurons [16], hence the CDFs of the marginals take the form
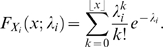


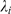
 is the mean spike-count of neuron 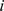 for a given bin size. A more flexible marginal is the negative binomial distribution,
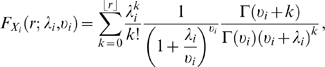
which allows to model spike-count distributions showing overdispersion. Here 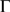 is the gamma function, 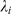 is again the mean spike-count of neuron 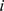, and 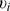 is a positive parameter, which controls the degree of overdispersion. The smaller the value of 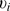, the greater is the Fano factor, and as 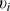 approaches infinity, the negative binomial distribution converges to the Poisson distribution.

**Figure 2:**
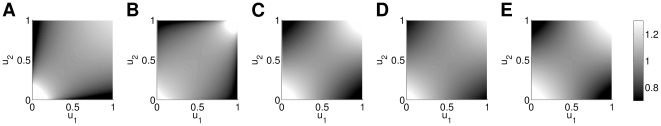
Bivariate copula probability densities of commonly used families. (A) Clayton copula (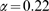). (B) Gumbel-Hougaard copula (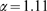). (C) Frank copula (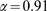). (D) Ali-Mikhail-Haq copula (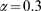). (E) Farlie-Gumbel-Morgenstern copula (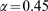).

The second part of the model is the copula family. Many different families have been discussed in the literature in the past. Families differ by the number of free parameters and by the class of dependence structures they can represent. The most simplistic copula is the product copula defined as 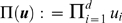 for which independence is attained. We selected a number of useful copula families (see Table 1). Figure 2 shows their bivariate probability density functions (PDFs).

**Table 1:** Five commonly used Copula families.

Copula Family	Cumulative Distribution Function 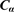	Constraints
Clayton	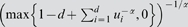	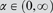
Gumbel-Hougaard	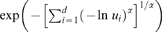	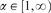
Frank	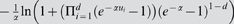	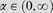
Ali-Mikhail-Haq	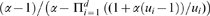	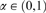
FGM	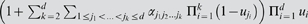	See caption^1^

Cumulative distribution functions of five copula families are listed. The parameter 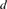 denotes the dimension of the distribution. 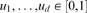 are the function arguments.

1 Constraints for the Farlie-Gumbel-Morgenstern family: 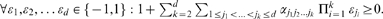

The Clayton family has a so-called lower tail dependence: the correlation between its elements is higher for low values than for high values (see Figure 2A). The scalar parameter 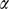 controls the strength of dependence. Note that 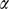 does not only control the strength of pairwise interactions but also the degree of higher order interactions. We define 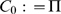.

The Gumbel-Hougaard (short Gumbel) family has an upper tail dependence. Here, the region of high correlation is in the upper right corner of the density. Hence, the correlation between its elements is higher for high values than for low values (see Figure 2B). The scalar parameter 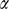 controls the strength of dependence.

The Frank family has no tail dependence. There is no difference between the correlation for low and for high values (see Figure 2C). Again, the scalar parameter 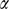 controls the strength of dependence and we define 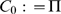.

The Ali-Mikhail-Haq (AMH) family models are positively ordered, i.e. for 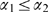 it holds for all 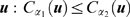 (see Figure 2D). Again we define 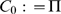.

The Farlie-Gumbel-Morgenstern (FGM) family has 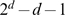 parameters that individually determine the pairwise and higher order interactions. It has 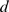 parameters less than the Poisson latent variables distribution because the rates of the neurons can be parametrized by the marginals. Non-zero values of the parameter 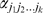 indicate the presence of 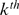 order interaction. For 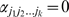 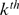 order interactions are absent. If, for example all 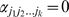 for 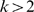, the corresponding probability distribution includes only parameters of second order, similar to the multivariate normal distribution. The constraints on the parameters 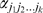, however, constrain the corresponding correlation to be small in terms of their absolute value.

### The Flashlight Transformation and Mixtures of Copulas

**Figure 3:**
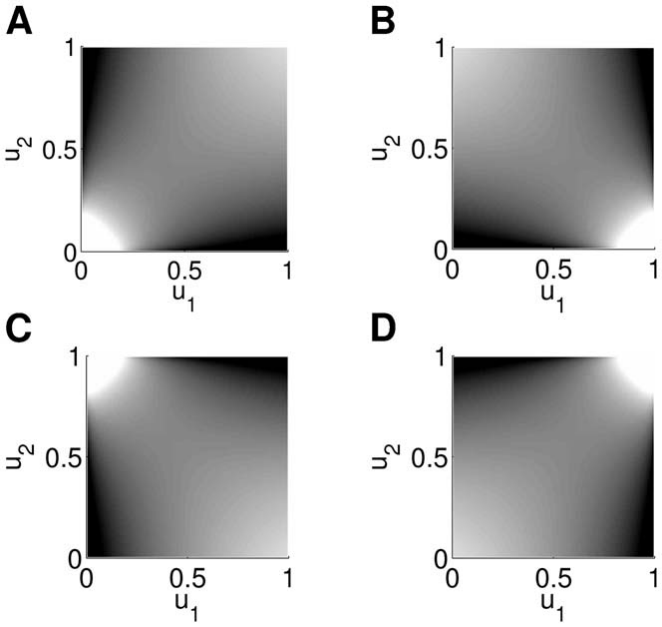
Probability densities of four different orthant dependencies generated by the flashlight transformation. The original distribution was the bivariate Clayton copula (parameter 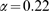). The transformation takes a set 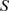 as a parameter which contains the indices of the elements that are transformed. (A) Original Clayton copula, which is also recovered for 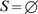. (B) Element 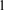 is transformed (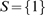). (C) Element 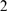 is transformed (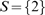). (D) Both elements are transformed (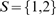).

We now introduce a novel extension of standard copula models, which is particularly useful for modeling distributions of spike-counts. It is based on the orthant dependence concept. Here, an *orthant* refers to one of the 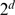 hypercubes of equal size in the unit hypercube, i.e. a “corner” of the copula distribution. Let us consider a distribution with a so-called lower tail dependence (see Figure 3A), i.e. a distribution, for which the correlation between spike-counts of two neurons is higher for low values than for high values. We now introduce the *flashlight transformation* which allows to shift the region of high correlation to an arbitrary orthant (see Figure 3B–D). The whole dependence structure between spike-counts is rotated accordingly, but remains unchanged otherwise. The transformation is a function that operates on CDFs. Yet, it rotates the corresponding PDF, not the CDF.

The flashlight transformation is specified in the following theorem (see Text S1 in the supplementary material):

#### Theorem 2

*Let* 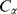 *be a d-copula,* 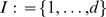 *,* 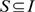 *,* 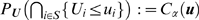 *a measure, and* 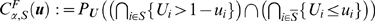 *. Then* 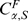 *is a d-copula and can be expressed as*

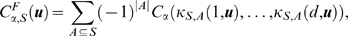
(1)where 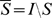 and 
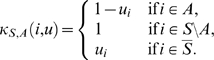


The flashlight transformation is a generalization of the so-called survival transformation, which is well known in the economics literature [17], and which is recovered for 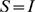. An example is shown in Figure 3D.

For heterogeneous data more versatile dependence structures are required. In order to generate this flexibility, one can construct finite mixtures of copulas each of which is weighted by a parameter 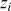 [18]. The CDF of mixtures of copulas takes the following form:
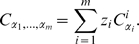



The latent variable 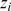 represents the responsibility of the corresponding copula 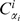.

### Model Fitting

Once a family of marginal distributions and a family of copulas for describing the dependence structure has been selected, model parameters have to be estimated from the data, i.e. from the empirical distribution. Here we suggest a method which is similar to maximum likelihood estimation.

Theorem 1 provides a method to construct multivariate CDFs based on copulas. Therefore, the approach yields a *CDF* of a multivariate distribution. In order to calculate the likelihood we have to transform the CDF to a probability mass function (PMF).

For this purpose we define the sets 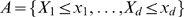 and 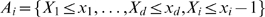, 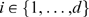. The probability of a particular set of spike-counts 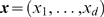 can then be expressed using only the CDF 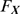, making use of the so-called inclusion-exclusion principle of Poincaré and Sylvester [19]:
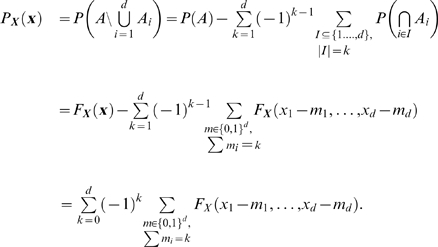
(2)


Let
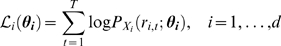
denote the sum of log likelihoods of the marginal distribution 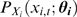, where 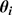 are the parameters of the chosen family of marginals. Furthermore, let
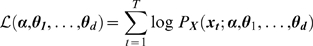
be the log likelihood of the joint probability mass function, where 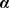 denotes the parameter of the chosen copula family. The so-called *inference for margins* (IFM) method [20] now proceeds in two steps. First, the marginal likelihoods are maximized separately:
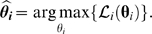



Then, the full likelihood is maximized given the estimated marginal parameters:
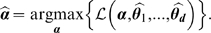



It was shown that the IFM estimator is asymptotically efficient [20]. The estimator is computationally more convenient than the maximum likelihood estimator, because parameter optimization in low dimensional parameter spaces needs less computation time.

Depending on whether the copula parameters are constrained, either the Nelder-Mead simplex method for unconstrained nonlinear optimization [21] or the line-search algorithm for constrained nonlinear optimization [22] can be applied to estimate the copula parameters using Eqn 2 as the objective function.

For mixtures of copulas, where the values of the latent variables 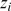 have to be estimated in addition, we suggest to use the expectation-maximization algorithm [23],[24]. In the expectation step, the weights 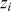 are updated using
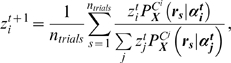
where 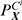 is the PMF of the model based on the copula 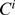. In the maximization step the copula parameters 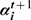 are determined for fixed values of 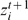 by applying the IFM method. Both steps are repeated until parameter values converge.

### Leaky Integrate-and-Fire Model for Generation of Synthetic Data

The leaky integrate-and-fire neuron is a simple neuron model that models only subthreshold membrane potentials. The equation for the membrane potential is given by
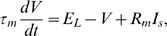
where 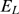 denotes the resting membrane potential, 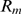 is the total membrane resistance, 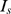 is the synaptic input current, and 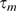 is the time constant. The model is completed by a rule which states that whenever 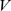 reaches a threshold 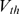, an action potential is fired and 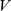 is reset to 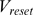 [25]. In all of our simulations we used 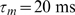, 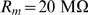, 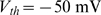, 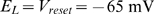, and initialized 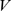 with 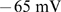. These are typical values that can be found in [25].

Current-based synaptic input for an isolated presynaptic release that occurs at time 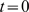 can be modeled by the so-called 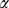-function [25]:
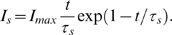



The function reaches its peak 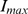 at time 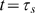 and then decays with time constant 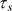. We can model an excitatory synapse by a positive 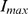 and an inhibitory synapse by a negative 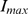. We used 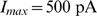 for excitatory synapses, 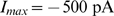 for inhibitory synapses, and 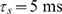.

### Multi-Tetrode Recordings

Neural activity was recorded from the lateral prefrontal cortex within an area of 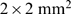 located on the ventral bank of the principal sulcus of an adult female rhesus monkey (*macaca mulatta*). Recordings were performed simultaneously from up to 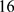 adjacent sites with an array of individually movable fiber micro-tetrodes (manufactured by Thomas Recording) with an inter-tetrode distance of 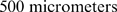. Data were sampled at 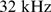 and bandpass filtered between 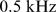 and 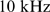. Recording positions of individual electrodes were chosen to maximize the recorded activity and the signal quality. The recorded data were processed by a principal component analysis-based spike sorting method. Automatic cluster cutting was manually corrected by subsequent cluster merging if indicated by quantitative criteria such as the ISI-histograms or amplitude stability.

Activity was recorded while the monkey performed a visual working memory task. One out of 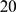 visual stimuli (fruits and vegetables) were presented for approximately 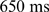. After a delay of 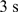, during which the monkey had to memorize the sample, a test stimulus (“test”) was presented and the monkey had to decide by differential button press whether both stimuli were the same or not. Correct responses were rewarded. Match and non-match trials were randomly presented with equal probability (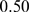).

#### Data preprocessing

We selected six neurons with stimulus specific responses, i.e. those neurons whose firing rate averaged over the time interval of presentation of the sample stimulus changed most compared to the pre-stimulus interval baseline. It turned out that each of these neurons was recorded from a different tetrode.

Spike trains were analyzed separately for each of the 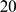 different stimuli and the four trial intervals: pre-stimulus, sample stimulus presentation, delay, and test stimulus presentation. Spike trains were binned into successive 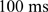 intervals and converted into six dimensional spike-counts for each bin. Due to the different interval lengths, the total sample size per condition varied between 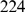 and 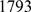. A representative example of the empirical distribution of a pair of these counts is presented in Figure 1A.

### Estimation of Mutual Information

The mutual information between spike-counts 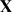 and stimuli is given by
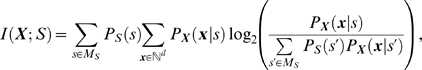
(3)where 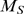 is the set of stimuli, 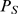 is the probability distribution over the stimuli, and 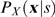 is the likelihood of a neural response 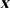 given a stimulus 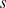. For higher dimensions 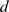 the sum over 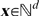 prohibits an exact computation of 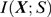, since the number of terms of the sum grows exponentially with 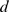. The evaluation of this sum is therefore practically infeasible unless the number of neurons is very small. However, we can estimate the mutual information using Monte Carlo sampling. For each of the stimuli 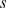, we can estimate the second sum by drawing samples 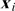 with probability 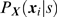. The term
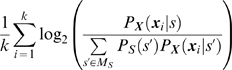
will then converge to the second sum in Eqn 3, as 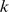 approaches infinity [26].

## Results

### Reliability of Model Estimation

**Figure 4:**
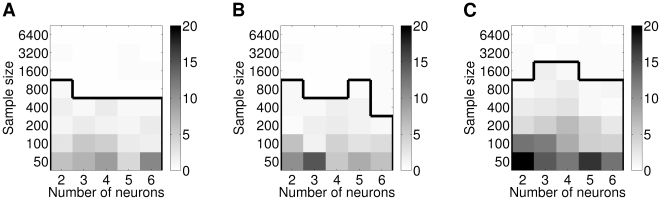
Deviation of the estimated likelihood from the likelihood for different dependence strengths. The deviation is given in percent of the likelihood. Samples were drawn from a Clayton-copula model with negative binomial marginals. The marginals were parametrized by maximum likelihood estimates obtained on the entire data that is described in Section “Multi-Tetrode Recordings”. The vertical axis indicates the number of samples in the training set. The evaluation took place on a separate set of 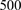 samples. Above the black line the deviation is smaller than 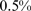. (A) Correlation coefficient 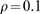. (B) Correlation coefficient 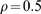. (C) Correlation coefficient 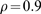.

Typically the number of samples that can be obtained in electro-physiological experiments is small. Thus, it might appear to be hopeless to estimate a multidimensional model with a detailed dependence structure. However, since our marginal distributions are discrete the copula matters only at a small number of points. In the following, we will demonstrate that it is not always necessary to obtain a great number of samples for a reliable model estimation. For this purpose we selected the Clayton-copula model with negative binomial marginals as a ground truth model which was used to draw samples. We calculated the deviation of the log likelihood of the estimated model from the log likelihood of the ground truth model in percent of the ground truth log likelihood. The correlation strength of the ground truth model was varied by the Clayton parameter. The results are shown in Figure 4 for three different Clayton parameters of the ground truth model. For moderate dependence strengths (as are typically found in the data) 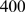 samples were sufficient for estimations of the log likelihood with an error of less than 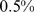.

### Application of Copula-Based Models to Synthetic Data

**Figure 5:**
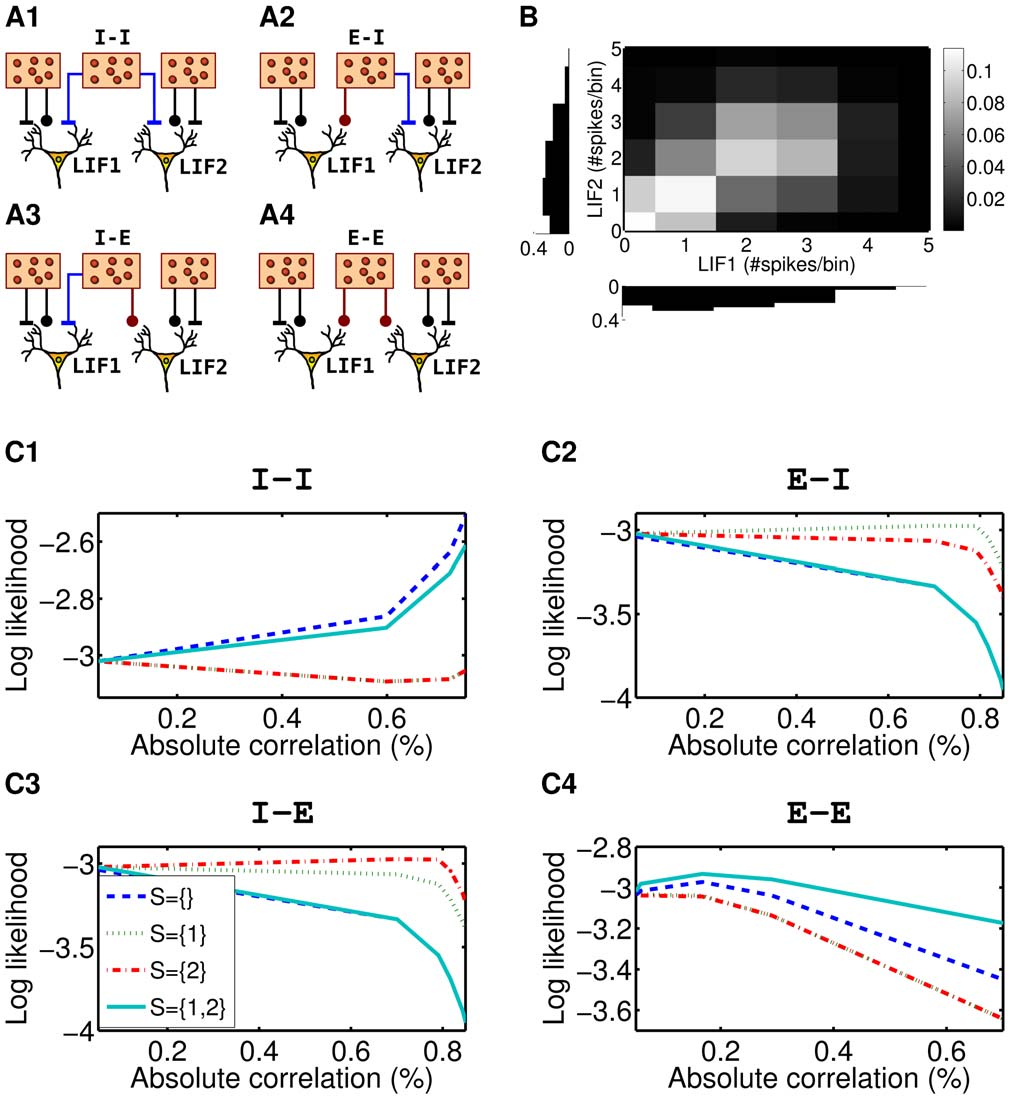
Copula-based analysis of bivariate spike-count data. (A1–A4) Neural network models used to generate the synthetic spike-count data. Two leaky integrate-and-fire neurons (“LIF1” and “LIF2”, see Section “Materials and Methods”) receive spike inputs from three separate populations of neurons (rectangular boxes and circles), but only one population sends input to both of the neurons. All input spike trains were Poisson-distributed. Each neuron had a total inhibitory input rate of 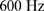. We had three times as many excitatory spikes as inhibitory spikes. We increased the absolute correlation between the spike-counts by shifting the rate of the left and right populations to the center population. The center population was active in half the simulation time. The total simulation time amounted to 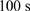. Spike-counts were calculated for 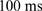 bins. (B) Empirical distribution for the model with an inhibitory input population (see A3) obtained for 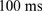 bins and a correlation coefficient of 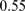. (C1–C4) Log likelihoods of the best fitting Clayton copulas with negative binomial marginals as a function of the strength of the input correlation. Plots shown (C1 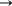 C4) correspond to the four different network models (A1 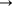 A4). Dotted, dashed, solid, and dashed-dotted lines correspond to the best fitting Clayton copula with lower, lower-right, upper-left, and upper orthant dependence (see Figure 3). Copulas were fitted using the IFM estimators.

One cause for dependence between spike-counts of different neurons are common input populations. Therefore, we investigated network models with different types of common input. We set up two current based leaky integrate-and-fire neurons (see Section “Materials and Methods”) and three input populations modeled as Poisson spike generators. The left input population projected only to neuron 1 and the right input population projected only to neuron 2. The center input population was the common input population, projecting to both neuron 1 and neuron 2. We investigated all four combinations of excitatory (E) and inhibitory (I) projections from the common population to the two neurons (see Figure 5A1–A4).

In this network model a lower tail dependence should arise if the projections from the common input projection are mostly inhibitory: each time the common population is active the firing rates of both neurons will decrease simultaneously. Therefore, only low spike-counts should be strongly correlated and the Clayton family should provide a good fit to the responses of such a network. Similarly, two excitatory projections should result in an upper tail dependence and other combinations should become apparent as dependence blobs in other corners of the probability density function of the copula. The flashlight transformation shifts the dependence blob of a given copula with orthant dependence into other orthants of the probability density function and is thus capable of modeling different types of common input populations in a stochastic manner. For two neurons, the lower left corner models an inhibitory input population, the upper right corner models an excitatory input population, and the other corners model a combination of excitatory and inhibitory input populations.

The spike trains of the two neurons were binned into 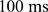 intervals. We applied copula-based models with negative binomial marginals to fit the generated data from the four models using the IFM method (see Section “Model Fitting”). Four different copula families were applied: the unmodified bivariate Clayton family and the three remaining flashlight transformations of the Clayton family (Figure 3). Figure 5C1–C4 shows the log likelihoods of the fits for the corresponding networks as shown in Figure 5A1–A4. The respective model performed best for the combination of projection types of the common input population it was supposed to model, i.e. Clayton for I-I, Clayton survival for E-E, etc. Hence, by determining the best fitting transformation the most likely combination of input types could be identified. Each of the transformations could be associated with a distinct combination of projection types.

To investigate whether the results of the reconstruction depend on the strengths of the synapses we varied 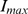 between 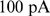 and 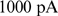 for excitatory synapses and between 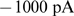 and 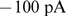 for inhibitory synapses (data not shown). While the relation of the best fitting copula families was constant across all strengths the differences between the curves decreased for decreasing strengths. For 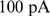 it was hard to distinguish between the likelihoods of lower and upper tail dependencies. Therefore, tail dependencies were less pronounced in the spike-counts. In the multi-tetrode data, however, we found significant differences between the likelihoods of the copula families (see Section “Application of Copula-Based Models to Multi-Tetrode Data”).

To investigate the impact of the bin size on the reconstruction performance we also binned the data into smaller and larger intervals (data not shown). When the bin size was too small or too large (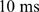 and 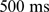) the reconstruction did not succeed. In the intermediate range (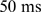, 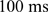), however, the connection types could be reconstructed. This can be explained by the asymptotic distributions of the multivariate spike-counts. According to the *central limit theorem* the multivariate normal distribution provides a good approximation when the bin size is sufficiently large. Hence, tail dependencies will vanish. On the contrary, when the bin size becomes too small the marginal distributions are essentially Bernoulli distributed and the tail dependencies will vanish as well. Of course, the range of the intermediate bin size depends on the rates of the neurons. The larger the rates the smaller the bin sizes in the intermediate range. For the simulated data the rates were comparable to the data recorded from the prefrontal cortex (see Section “Multi-Tetrode Recordings”).

### Application of Copula-Based Models to Multi-Tetrode Data

Our copula-based models are capable of modeling different dependence structures with marginals that are tailored to single neuron spike-count distributions. Thus, we expected that the copula-based models would provide a much better fit to data recorded from real neurons than the multivariate normal distribution or the multivariate Poisson latent variables distribution. To test this, we applied copula-based models from different families and with different marginal distributions to data, which has been recorded from macaque prefrontal cortex for each of the twenty presented stimuli and each of the four phases (pre-stimulus presentation, stimulus presentation, delay, presentation of the test stimulus) of the visual working memory task. We compared the results to models of the discretized multivariate normal and the Poisson latent variables distribution (see Section “Materials and Methods”)

We randomly selected 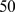 count vectors for each task phase and each stimulus as the validation set. We then estimated the model parameters on the remaining count vectors (training set) and used the validation set for obtaining an unbiased estimate of the likelihoods of the selected models.

We used the IFM-estimator for the copula-based models and the maximum likelihood estimator for the Poisson latent variables distribution. The parameters 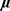 and 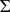 of the discretized MVN distribution were estimated by the sample mean and the sample covariance matrix of the spike-counts. This procedure does not correspond to the maximum likelihood estimate of the discretized distribution. We used it, because the maximum likelihood estimator was too expensive to compute for six neurons. The high computational costs come from the estimation of the CDF of the MVN.

The rate parameter 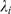 for the Poisson distribution and negative binomial distribution were estimated via the sample mean. The maximum likelihood estimates for the overdispersion parameter 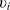 were computed iteratively by Newton's method.

**Figure 6:**
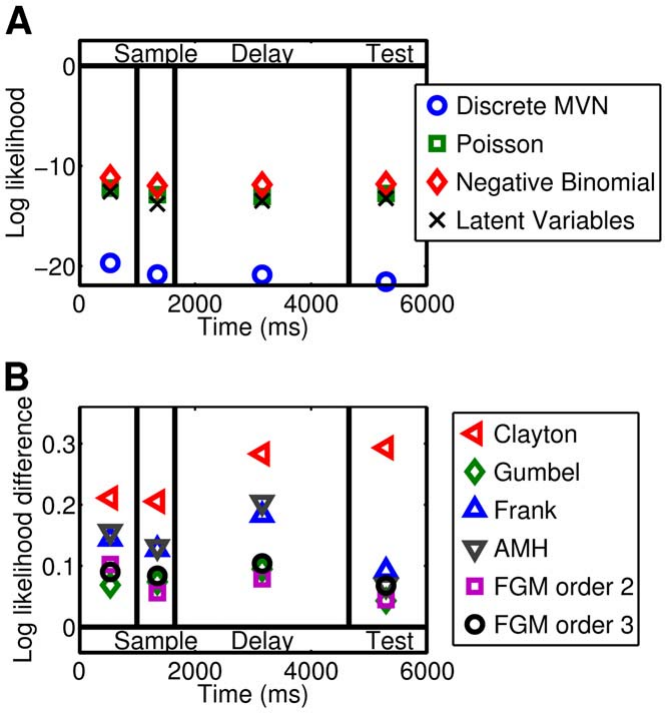
Log likelihoods of the best fitting MVN, Poisson latent variables, and copula-based models for the validation set. (A) Log likelihoods for the discretized multivariate normal distribution (circles), the multivariate Poisson latent variables distribution (crosses), the best fitting copula-based model with Poisson (squares), and with negative binomial marginals (diamonds). The figure shows the log likelihoods averaged over all 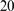 different stimuli, but separately for the pre-stimulus, sample stimulus, delay, and test stimulus phase of the memory task. For the best fitting copula, we considered all the copula families shown in B. AMH denotes the Ali-Mikhail-Haq family, FGM the Farlie-Gumbel-Morgenstern family (see Table 1). For the 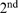 order model of the FGM family we set all but the first 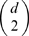 parameters to zero, therefore leaving only parameters for pairwise interactions. In contrast, for the 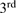 order model we set all but the first 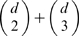 parameters to zero. (B) Difference between the log likelihood of a model with independent spike-counts and negative binomial marginals (“ind. model”) and the log likelihoods of the best fitting representatives of the different copula-based models shown in the legend. Negative binomial marginals were used. Data was again averaged over the 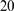 different stimuli.

Figure 6A summarizes the results for the discretized MVN, the Poisson latent variables distribution, and two copula-based distributions with different marginals, the Poisson distribution, and the negative binomial distribution. The negative binomial distribution provided for all four task phases a significantly better fit than the Poisson distribution, the MVN distribution, and the Poisson latent variables distribution. The likelihood for the copula-based models was significantly greater than for the discretized MVN model (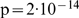, paired-sample Student's *t* test over stimuli) and the Poisson latent variables model (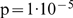). Moreover, the likelihood for the negative binomial marginals was even greater than that for the Poisson marginals (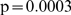). Thus, the copula-based approach provided models that were indeed superior for the data at hand. Moreover, the additional flexibility of the negative binomial marginals improved the fit significantly.

We applied different copula families to examine the importance of the dependence structure for the model fit. Figure 6B shows an evaluation of the different copula families with different dependence structures for the best fitting marginal, which was the negative binomial distribution. The model based on the Clayton copula family provided the best fit. The fit was significantly better than for the second best fitting copula family (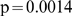), the Gumbel family. In spite of having more parameters, the FGM copulas performed worse. However, the FGM model with third order interactions fitted the data significantly better than the model that included only pairwise interactions (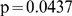).

**Figure 7:**
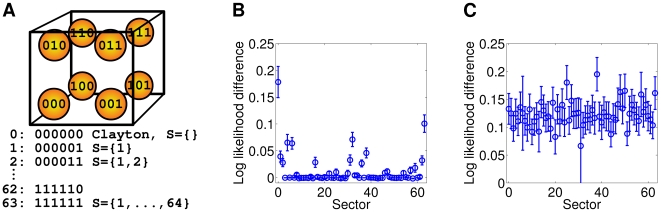
Log likelihoods of different Clayton-copula models transformed using the flashlight transformation. (A) Cartoon indicating the labeling of orthants for the six dimensional space. Each number indicates the orthant, into which the originally lower tail dependence was transformed. (B) Mean log likelihoods on the test interval validation set for all possible flashlight transformed Clayton copulas and negative binomial marginals. The bars mark the standard errors. (C) Mean log likelihoods on the test interval validation set for a mixture of the Clayton copula with all possible flashlight transformed Clayton copulas and negative binomial marginals.

The best fitting copula-based model, the Clayton copula, is characterized by a lower tail dependence. Apart of the Gumbel family, the other families that we applied so far do not model orthant dependencies. To check whether other orthant dependencies would improve the fit, we applied the flashlight transformation and we transformed the Clayton copula tail towards all corners of the six dimensional hyper cube. The results are shown in Figure 7. The standard Clayton copula with lower tail dependence had the significantly highest value of the log likelihood on the validation set indicating that the empirical spike-count distribution has indeed a lower tail dependence. The second highest peak was reached by the Clayton survival copula. The central peak corresponded to those transformations that were close to the Clayton and the Clayton survival copulas: sectors 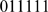 and 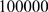 (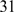 and 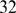 decimal). Thus, a common lower tail dependence was prominent in the data.

We applied mixtures of copulas as described in Section “The Flashlight Transformation and Mixtures of Copulas” to check whether there was indeed a prominent common upper tail dependence beside the lower tail dependence in the data. Therefore, we fixed the Clayton copula (which models a lower tail dependence) as the first mixture component and varied the sector of the flashlight transformed Clayton copula for the second mixture component. Figure 7C shows the mean log likelihoods of the mixture models with negative binomial marginals on the same data set used for Figure 7B. All of the mixture models exhibit similar performance. Therefore, the upper tail dependence that we observed for the unmixed model appears to be an artifact of the lower tail dependence.

In summary, we could show that the copula-based approach provided a significant improvement in the goodness of fit compared to the discretized and rectified multivariate normal distribution and the Poisson latent variables distribution. Moreover, the dependence structure alone has a significant impact as well.

### Appropriateness of Model

Our model consists of two parts: 1) the copula and 2) the marginals. We already analyzed the effect of the copula. In this section we describe the investigation of the marginals. In particular, we are interested in understanding how the goodness of fit is influenced by the marginals. For this purpose we compared the log likelihoods of the Clayton-copula model with Poisson, negative binomial, and empirical marginals fitted to the training set of the sample stimulus presentation phase. The model with empirical marginals was a so-called semiparametric distribution consisting of a parametric dependence structure (the copula family) and nonparametric marginals. We drew samples from these distributions in order to learn whether the training and validation sets were typical samples from the fitted distributions. For a complex model we expect the likelihood of training samples to be close to the mode of the histogram, while we expect the validation samples to have a much smaller likelihood. Contrary, for a model with small complexity we expect the likelihood of the training samples to be close to the likelihood of the validation samples. When the complexity is too small we expect the likelihoods of the training and the validation samples to be much smaller than the mode of the histogram.

**Figure 8:**
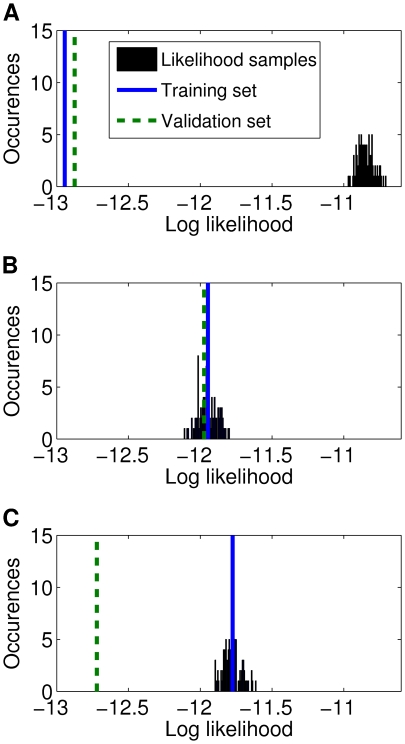
Distribution of log likelihoods from models fitted to data from the sample stimulus phase. The Clayton-copula model with different marginals was used. A histogram of 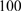 samples is shown where each sample represents an average over 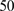 spike-count vectors. The solid line corresponds to the log likelihood of the training set whereas the dashed line corresponds to the log likelihood of the validation set. (A) Model with Poisson marginals. (B) Model with negative binomial marginals. (C) Model with empirical marginals.

In our setting the most complex model is the one with empirical marginals. Histograms of the log likelihoods for copula models with the three different marginals are shown in Figure 8. For Poisson marginals, the log likelihoods of both the training set and the validation set were much smaller than the log likelihoods of the samples drawn from the fitted distribution. Thus, the Poisson marginals seem to be too simple for a good fit to the data, whereas the negative binomial marginals generalized well in spite of their increased complexity. On the training set the model with the empirical marginals performed best. However, there was a huge discrepancy to the likelihood of the model with empirical marginals on the validation set, whereas the likelihoods of the other two models did not change much. This result can be explained by overfitting. The empirical marginals matched the marginals of the training set perfectly. The empirical marginals of the training set, however, were noisy representations of the true marginals, because of the limited sample size. Hence, a perfect fit is not beneficial when it comes to novel data. In contrast to that, the likelihoods of the models with Poisson and negative binomial marginals were almost equal to the respective likelihoods on the training set. Thus, these models did not suffer from overfitting.

In order to relate these findings to the number of samples in our training set we can compare the number of samples to the estimated number of required samples for the toy example in Section “Model Fitting”. Figure 6 shows that the log likelihood for the Clayton-copula model deviated from the second best family by 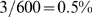. In Section “Model Fitting” we showed that for this model 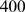 samples were sufficient for good estimations of the log likelihood. For the delay phase and for the test stimulus phase, the number of samples varied between 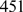 and 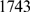 per stimulus. Therefore, the number of samples was sufficient for these phases. Taken together with the histogram analysis, we found that the model complexity was appropriate for the available amount of data at hand.

### Information Analysis

**Figure 9:**
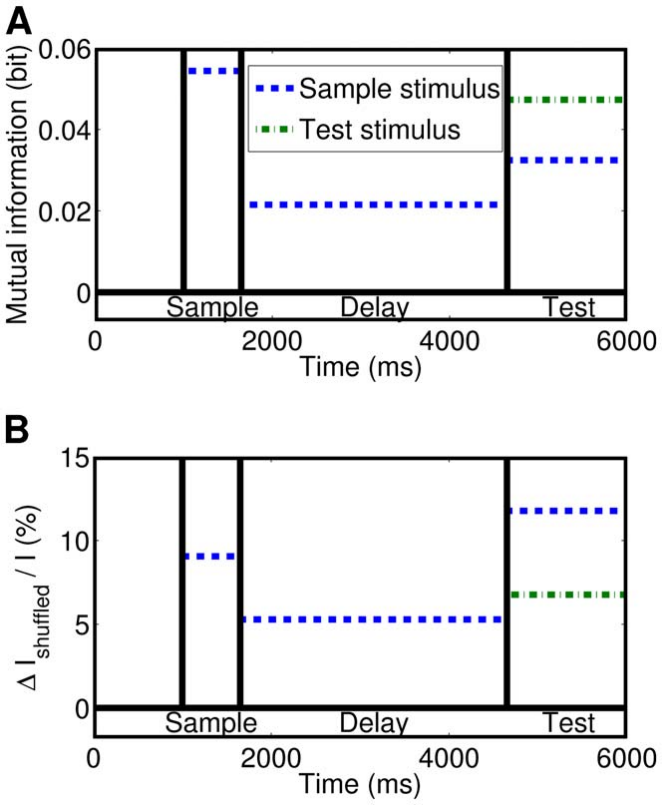
Monte Carlo estimates of the mutual information between stimuli and responses. The estimation is based on the Clayton-copula model with negative binomial marginals. The Monte Carlo method was terminated when the standard error was below 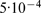. The sample stimulus was presented in phase two, whereas the test stimulus was presented in phase four. For the test stimulus phase, the estimation was performed twice: for the sample stimulus that was previously presented (dashed line) and for the test stimulus (dotted line). (A) Estimated mutual information based on IFM parameters determined on the training set for each of the task phases (pre-stimulus, sample stimulus, delay, and test stimulus). (B) Estimated information increase that is due to the dependence structure. The mutual information 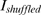 of the model with independent spike-counts and negative binomial marginals was subtracted from and normalized to the mutual information 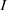 of the Clayton-copula model with negative binomial marginals.

We will now show that the copula-based models can be used to measure the short-term information about a stimulus that is encoded by the spike-count dependence structure of the recorded neurons. The first step is to estimate the total information of the spike-count responses. We applied the best fitting copula model, the Clayton-copula model with negative binomial marginals, to estimate the mutual information between stimuli and responses via Monte Carlo sampling (see Section “Materials and Methods”). Figure 9A shows the estimated mutual information for each of the four task phases. The mutual information was greater during the sample stimulus interval and the test stimulus interval than during the delay interval. Therefore, a stimulus presentation evoked a spike-count response which instantly encoded information about the stimulus. In the test stimulus phase the dotted line is above the dashed line, so the spike-counts coded more information about the sample stimulus that was previously presented than about the test stimulus.

Figure 9B shows the information estimate 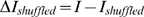, normalized to the mutual information 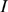 that is shown in Figure 9A. The dependence structure carried between 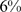 and 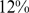 of the mutual information. During the test stimulus interval the dependence structure encoded almost twice as much information about the test stimulus as about the sample stimulus that was previously presented.

## Discussion

We developed a framework for analyzing the noise dependence of spike-counts and used synthetic data from a model of leaky integrate-and-fire neurons to derive interpretations for different dependence structures. Applying the framework to our data from the macaque prefrontal cortex we found that: (1) copula-based models with negative binomial marginals rather than the multivariate normal distribution or the Poisson latent variables distribution are appropriate models of spike-count data for short time intervals; (2) the dependence structure encodes between 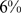 and 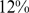 of the mutual information about the presented stimuli; (3) the amount of data required for a good likelihood estimation is present in our data set; and (4) a lower tail dependence between all neurons is present in the data and can be explained by common inhibitory input.

The copula approach has many advantages compared to previous models. Recently, the Ising model gained a lot of attention in neuroscience [4],[27]. This model is a maximum entropy model of binary variables called spins that have only pairwise interactions [28]. The model is applied to the neuroscience setting by binning spike trains into very short time intervals such that at most one spike falls into each bin. The spin for that bin then indicates whether or not a spike was present. Using this model pairwise interactions between simultaneously recorded neurons can be modeled [4]. The Ising model is a special case of a more general class of nested maximum entropy models [29]. Other models in this class can be used to model higher order interactions between neurons. Nevertheless, an independence assumption for subsequent bins is necessary due to the limited number of samples present in typical neuroscience settings. Therefore, the marginal spike-counts of individual neurons will be binomial distributed. The variance of this distribution is always smaller than its mean which is a severe disadvantage of this model class. The copula approach on the other hand can model arbitrary marginals.

Another class of models are doubly stochastic models where some parameters of the data distribution are themselves random variables. The doubly stochastic Poisson point process presented by Krumin and Shoham belongs to this class [30]. For such models the marginal distributions change whenever the dependence is modified. It is thus very hard to disentangle the effects of the dependence structure from the effects of the marginals.

In contrast to the multivariate normal distribution and the multivariate Poisson latent variables distribution the copula approach can be used to model arbitrary marginal distributions that are appropriate for the data at hand. The marginal distributions can therefore be discrete without any mass on the negative axis and with variance greater than the mean. We compared the fits of negative binomial marginals to Poisson and empirical marginals and found that only the negative binomial marginals provided a reasonable fit to the data. Contrary to the Poisson marginals, the negative binomial marginals were complex enough such that likelihoods of samples from the model were consistent with the likelihood of the data. Moreover, the negative binomial marginals did not suffer from overfitting as did the empirical marginals. We conclude that the negative binomial marginals are appropriate to describe the spike-counts recorded from the prefrontal cortex.

The dependence structure of the copula approach is flexible. Higher order interactions can be parametrized separately if desired. Furthermore, in contrast to the multivariate Poisson latent variables distribution, negative correlations can be modeled as well. Another advantage of the copula approach is that it is modular in the sense that the copula family used for the data analysis can be easily exchanged by another family. Many different copula families exist, each representing and parameterizing different properties of the dependence structures. Thus, it is easy to test for different properties of a distribution. Specific examples are the Clayton and Gumbel families. These families have lower and upper tail dependencies, respectively. Lower and upper tail dependencies can arise from common input populations with inhibitory and excitatory projections, respectively. By deriving the flashlight transformation we could construct additional families that account for combinations of inhibition and excitation.

When applying the flashlight transformation to the data from the prefrontal cortex, we found that the unmodified Clayton family provided the best fit to the test data. Therefore, a common lower tail dependence to all neurons is present in the data. One explanation is a common input population whose projections are mostly inhibitory to all the analyzed neurons. Two types of common inhibitory sources are possible: (1) A local source of inhibitory input such as common interneurons. (2) Another area projecting to the prefrontal cortex. It was found that interneurons have a reach of no more than a few hundred micrometers whereas the inter-tetrode distance was 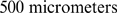. Thus, it is unlikely that a population of common interneurons inhibits all the stimulus specific neurons that we recorded from. Another area, therefore, is more likely to be the source of the common inhibitory input. One possibility could be the ventral tegmental area (VTA). In the rat cortex it was found that the VTA exerts a direct inhibitory influence on the PFC. In a study 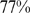 of 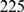 recorded PFC neurons were inhibited as a result of VTA stimulation [31]. Moreover, the VTA is thought to be a central component of the reward system [32] which is essential for a memory task. Our analysis provides evidence for such an influence based on the spike-count statistics.

The second best fit was achieved by the Clayton survival family. One explanation for this result is provided by an upper tail dependence between all neurons in addition to the stronger lower tail dependence. We applied mixtures of copulas to elucidate this issue and found that a mixture of the Clayton and Clayton survival family did not provide the best fit out of all mixtures of the Clayton family with a Clayton flashlight transformation. At first sight it is puzzling that the upper tail dependence seems to disappear when mixed with the lower tail dependence. However, the Clayton copula and the Clayton survival copula have their dependence along the same line in the six dimensional space that is spanned by the neuronal spike-counts, though predominantly at different ends of this line. Hence, the Clayton survival family can capture some of the dependence that is inherent to the Clayton family. We conclude that the prominence of the upper tail dependence that was observed for the unmixed model is an artifact of the lower tail dependence component.

The results show that important properties of dependence structures such as tail dependencies arise very naturally in simple input scenarios, and that the copula approach can be used to construct generative models that are capable of capturing these aspects of this underlying connectivity. In principle, copula-based models can be used to guide reconstructions of functional connectivity, but this topic is outside the scope of this study. If the reader is interested in detailed reconstruction of functional connectivity we recommend the studies in [33]–[35] as a starting point.

We could show that there is important information represented in the dependence structure which has been ignored in studies reporting only the correlation coefficient. Based on the flashlight transformation we could derive novel copula families with interesting interpretations for neuroscience: the statistical dependence gives insight into possible connections of the underlying network. Other copula families might be applicable to investigate different properties of the network.

We could also show that the Gaussian distribution is not an appropriate approximation of the spike-count distribution of short time intervals. Yet, many studies applied this approximation in their investigations. Therefore, these studies should be reassessed with respect to their validity for short-term coding.

We also compared the copula-based approach to the multivariate Poisson latent variables distribution. In terms of spike-counts this model corresponds to previous point process models that account for higher order correlations. The copula-based approach overcomes a number of shortcomings of this distribution, namely the Poisson marginals, the restriction to non-negative correlations and the inflexible dependence structure. We could show that the improvement in the goodness-of-fit is significant.

Taken together, the copula-based approach allows us to model and analyze spike-count dependencies in much more detail than previously applied models. A drawback is the small number of neurons to which the approach can be applied so far. The approach is computationally too demanding for higher numbers of neurons because the model fitting complexity is exponential in the number of neurons. Approximate inference methods might provide a solution to the computational problem. However, another problem is the number of samples available in typical electro-physiological experiments. We could show that 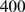 samples are sufficient for six dimensional data with moderate dependence strengths. Nevertheless, the amount of required data increases dramatically for increasing dimensions, i.e. for the number of neurons. A combination with dimensionality reduction techniques might provide a solution to this problem.

## Supporting Information

Text S1 Proof of the theorem that introduces the flashlight transformation for copula families.
(0.08 MB PDF)
