# Supplementary material for: Analyzing Short-Term Noise Dependencies of Spike-Counts in Macaque Prefrontal Cortex Using Copulas and the Flashlight Transformation
Source: PLoS Comput Biol. 2009 Nov 26;5(11):e1000577. doi: 10.1371/journal.pcbi.1000577 (PMC2776173; doi:10.1371/journal.pcbi.1000577)
Supplement: Text S1 — Proof of the theorem that introduces the flashlight transformation for copula families. (0.08 MB PDF) [file pcbi.1000577.s001.pdf]

Text S1

We prove Theorem 2 which states:

Theorem 2. *Let C_α be a d-copula, $I := \{1, \dots, d\}$, $S \subseteq I$, $P_{\boldsymbol{U}}(\bigcap_{i\in S}\{U_i \le u_i\}) := C_\alpha(\boldsymbol{u})$ a measure, and $C^F_{\alpha,S}(\boldsymbol{u}) := P_{\boldsymbol{U}}\left(\left(\bigcap_{i\in S}\{U_i > 1-u_i\}\right) \cap \left(\bigcap_{i\in \overline{S}}\{U_i \le u_i\}\right)\right)$. Then $C^F_{\alpha,S}$ is a d-copula and can be expressed as*

$$C^F_{\alpha,S}(\boldsymbol{u}) = \sum_{A\subseteq S}(-1)^{|A|}C_\alpha(\kappa_{S,A}(1,\boldsymbol{u}),\dots,\kappa_{S,A}(d,\boldsymbol{u})), \tag{1}$$

where $\overline{S} = I \setminus S$ *and* $\kappa_{S,A}(i,\boldsymbol{u}) = \begin{cases} 1-u_i & \text{if } i \in A, \\ 1 & \text{if } i \in S\setminus A, \\ u_i & \text{if } i \in \overline{S}. \end{cases}$

We need a short lemma for the proof:

Lemma 3. *Let $(\Omega, \mathcal{F}, P)$ be a probability space with $A \in \mathcal{F}$. Then $P^A := P(\bullet \cap A)$ is a measure on $\mathcal{F}$.*

Proof. Verification of the measure properties:

1. $P^A(\emptyset) = P(\emptyset \cap A) = P(\emptyset) = 0$.
2. For $E_1, E_2, \dots \in \mathcal{F}$ pairwise disjoint:

$$P^A\left(\bigcup_{i=1}^\infty E_i\right) = P\left(\left(\bigcup_{i=1}^\infty E_i\right)\cap A\right) = P\left(\bigcup_{i=1}^\infty (E_i\cap A)\right) = \sum_{i=1}^\infty P(E_i\cap A) = \sum_{i=1}^\infty P^A(E_i).$$

□

Proof of the Main Theorem. We start with Eqn 1. By definition $P_{\boldsymbol{U}}$ is a probability mass function. Let $B := \left(\bigcap_{i\in\overline{S}}\{U_i \le u_i\}\right)$. We can apply the generalized inclusion exclusion principle for arbitrary measures [1]:

$$\begin{aligned}
C^F_{\alpha,S}(\boldsymbol{u}) &= P^B_{\boldsymbol{U}}\left(\bigcap_{i\in S}\{U_i > 1-u_i\}\right) = P^B_{\boldsymbol{U}}(B) - P^B_{\boldsymbol{U}}\left(\overline{\bigcap_{i\in S}\{U_i > 1-u_i\}}\right)\\
&= P^B_{\boldsymbol{U}}(B) - P^B_{\boldsymbol{U}}\left(\bigcup_{i\in S}\{U_i \le 1-u_i\}\right)\\
&\overset{\substack{Lemma\ 3\\ Sieve}}{=} P^B_{\boldsymbol{U}}(B) - \sum_{k=1}^{|S|}(-1)^{k-1}\sum_{\substack{A\subseteq S\\|A|=k}} P^B_{\boldsymbol{U}}\left(\bigcap_{i\in A}\{U_i \le 1-u_i\}\right)\\
&= \sum_{k=0}^{|S|}(-1)^{k}\sum_{\substack{A\subseteq S\\|A|=k}} P^B_{\boldsymbol{U}}\left(\bigcap_{i\in A}\{U_i \le 1-u_i\}\right)\\
&= \sum_{A\subseteq S}(-1)^{|A|}P^B_{\boldsymbol{U}}\left(\bigcap_{i\in A}\{U_i \le 1-u_i\}\right)\\
&= \sum_{A\subseteq S}(-1)^{|A|}P_{\boldsymbol{U}}\left(\left(\bigcap_{i\in A}\{U_i \le 1-u_i\}\right)\cap\left(\bigcap_{i\in\overline{S}}\{U_i \le u_i\}\right)\right)\\
&= \sum_{A\subseteq S}(-1)^{|A|}C_\alpha(\kappa_{S,A}(1,\boldsymbol{u}),\dots,\kappa_{S,A}(d,\boldsymbol{u})).
\end{aligned}$$

Now we will show that $C^F_{\alpha,S}$ is a copula. We verify the copula properties:

1. Let $\boldsymbol{u} \in [0,1]^d$ with $\exists a \in I : u_a = 0$. We distinguish two cases:
   - For $a \in \overline{S}$:

$$\begin{aligned} C^F_{\alpha,S}(\boldsymbol{u}) &= \sum_{A\subseteq S} (-1)^{|A|} C_\alpha(\kappa_{S,A}(1,\boldsymbol{u}),\dots,\kappa_{S,A}(a-1,\boldsymbol{u}),0,\kappa_{S,A}(a+1,\boldsymbol{u}),\dots,\kappa_{S,A}(d,\boldsymbol{u})) \\ &= \sum_{A\subseteq S} (-1)^{|A|} 0 = 0. \end{aligned}$$

   - For $a \in S$:

$$\begin{aligned} C^F_{\alpha,S}(\boldsymbol{u}) &= \sum_{A\subseteq S\setminus\{a\}} (-1)^{|A\cup\{a\}|} C_\alpha(\kappa_{S,A\cup\{a\}}(1,\boldsymbol{u}),\dots,\kappa_{S,A\cup\{a\}}(a-1,\boldsymbol{u}),1,\kappa_{S,A\cup\{a\}}(a+1,\boldsymbol{u}),\dots, \\ &\quad \kappa_{S,A\cup\{a\}}(d,\boldsymbol{u})) + \sum_{A\subseteq S\setminus\{a\}} (-1)^{|A|} C_\alpha(\kappa_{S,A}(1,\boldsymbol{u}),\dots,\kappa_{S,A}(a-1,\boldsymbol{u}),1,\kappa_{S,A}(a+1,\boldsymbol{u}),\dots,\kappa_{S,A}(d,\boldsymbol{u})) \\ &= \sum_{A\subseteq S\setminus\{a\}} (-1)^{|A|+1} C_\alpha(\kappa_{S,A}(1,\boldsymbol{u}),\dots,\kappa_{S,A}(a-1,\boldsymbol{u}),1,\kappa_{S,A}(a+1,\boldsymbol{u}),\dots,\kappa_{S,A}(d,\boldsymbol{u})) \\ &\quad + \sum_{A\subseteq S\setminus\{a\}} (-1)^{|A|} C_\alpha(\kappa_{S,A}(1,\boldsymbol{u}),\dots,\kappa_{S,A}(a-1,\boldsymbol{u}),1,\kappa_{S,A}(a+1,\boldsymbol{u}),\dots,\kappa_{S,A}(d,\boldsymbol{u})) \\ &= 0. \end{aligned}$$

2. Let $\boldsymbol{u} \in [0,1]^d$ with $u_i = 1$ if $i \neq a$. We distinguish the same cases:
   - For $a \in \overline{S}$:

$$\begin{aligned} C^F_{\alpha,S}(\boldsymbol{u}) &= (-1)^{|\emptyset|} C_\alpha(\kappa_{S,\emptyset}(1,\boldsymbol{u}),\dots,\kappa_{S,\emptyset}(d,\boldsymbol{u})) + \sum_{\substack{A\subseteq S\\A\neq\emptyset}} (-1)^{|A|} C_\alpha(\kappa_{S,A}(1,\boldsymbol{u}),\dots,\kappa_{S,A}(d,\boldsymbol{u})) \\ &\overset{(1)}{=} (-1)^0 C_\alpha(\kappa_{S,\emptyset}(1,\boldsymbol{u}),\dots,\kappa_{S,\emptyset}(a-1,\boldsymbol{u}),\kappa_{S,\emptyset}(a,\boldsymbol{u}),\kappa_{S,\emptyset}(a+1,\boldsymbol{u}),\dots,\kappa_{S,\emptyset}(d,\boldsymbol{u})) \\ &\overset{(2)}{=} C_\alpha(\underbrace{1,\dots,1}_{a-1 \text{ times}},u_a,\underbrace{1,\dots,1}_{d-a \text{ times}}) = u_a. \end{aligned}$$

(1) $\forall A \subseteq S, A \neq \emptyset : a \in \overline{S} \Rightarrow a \notin A \Rightarrow \exists i \in A : i \neq a$, thus $\kappa_{S,A}(i,\boldsymbol{u}) = 1 - u_i \overset{i\neq a}{=} 0$. Therefore, we have:

$$\begin{aligned} &\sum_{\substack{A\subseteq S\\A\neq\emptyset}} (-1)^{|A|} C_\alpha(\kappa_{S,A}(1,\boldsymbol{u}),\dots,\kappa_{S,A}(d,\boldsymbol{u})) \\ &= \sum_{\substack{A\subseteq S\\A\neq\emptyset}} (-1)^{|A|} C_\alpha(\kappa_{S,A}(1,\boldsymbol{u}),\dots,\kappa_{S,A}(i-1,\boldsymbol{u}),0,\kappa_{S,A}(i+1,\boldsymbol{u}),\dots,\kappa_{S,A}(d,\boldsymbol{u})) \\ &= \sum_{\substack{A\subseteq S\\A\neq\emptyset}} (-1)^{|A|} 0 = 0. \end{aligned}$$

(2) $\kappa_{S,\emptyset}(a,\boldsymbol{u}) \overset{a\in\overline{S}}{=} u_a;\quad \forall i \in S : \kappa_{S,\emptyset}(i,\boldsymbol{u}) = 1;\quad \forall i \in \overline{S}\setminus\{a\} : \kappa_{S,\emptyset}(i,\boldsymbol{u}) = 1.$

   - For $a \in S$:

$$\begin{aligned} C^F_{\alpha,S}(\boldsymbol{u}) &= (-1)^{|\emptyset|} C_\alpha(\kappa_{S,\emptyset}(1,\boldsymbol{u}),\dots,\kappa_{S,\emptyset}(d,\boldsymbol{u})) \\ &\quad +(-1)^{|\{a\}|} C_\alpha(\kappa_{S,\{a\}}(1,\boldsymbol{u}),\dots,\kappa_{S,\{a\}}(d,\boldsymbol{u})) \\ &\quad + \sum_{\substack{A\subseteq S\\A\neq\emptyset, A\neq\{a\}}} (-1)^{|A|} C_\alpha(\kappa_{S,A}(1,\boldsymbol{u}),\dots,\kappa_{S,A}(d,\boldsymbol{u})) \\ &\overset{(3)}{=} C_\alpha(\kappa_{S,\emptyset}(1,\boldsymbol{u}),\dots,\kappa_{S,\emptyset}(d,\boldsymbol{u})) - C_\alpha(\kappa_{S,\{a\}}(1,\boldsymbol{u}),\dots,\kappa_{S,\{a\}}(d,\boldsymbol{u})) \\ &\overset{(4)}{=} C_\alpha(\underbrace{1,\dots,1}_{d \text{ times}}) - C_\alpha(\kappa_{S,\{a\}}(1,\boldsymbol{u}),\dots,\kappa_{S,\{a\}}(d,\boldsymbol{u})) \end{aligned}$$

$$\stackrel{(5)}{=} \quad 1 - C_\alpha(\underbrace{1,\dots,1}_{a-1 \text{ times}}, 1-u_a, \underbrace{1,\dots,1}_{d-a \text{ times}})$$
$$= \quad 1-(1-u_a) = u_a.$$

(3) $\forall A \subseteq S, A \neq \emptyset, A \neq \{a\} : \exists i \in A : i \neq a$, remainder as in (1).

(4) $\kappa_{S,\emptyset}(a,\boldsymbol{u}) = 1; \quad \forall i \in S \setminus \{a\} : \kappa_{S,\emptyset}(i,\boldsymbol{u}) = 1; \quad \forall i \in \overline{S} : \kappa_{S,\emptyset}(i,\boldsymbol{u}) = 1.$

(5) $\kappa_{S,\{a\}}(a,\boldsymbol{u}) = 1-u_a; \quad \forall i \in S \setminus \{a\} : \kappa_{S,\{a\}}(i,\boldsymbol{u}) = 1; \quad \forall i \in \overline{S} : \kappa_{S,\{a\}}(i,\boldsymbol{u}) = u_i = 1.$

3. We show that $C^F_{\alpha,S}$ is d-increasing, i.e. $\forall B = \times_{i=1}^d [u_i, v_i] : \mathrm{V}_{C^F_{\alpha,S}}(B) \geq 0$,
where $\mathrm{V}_{C^F_{\alpha,S}}(B) = \sum_{i_1=1}^2 \cdots \sum_{i_d=1}^2 (-1)^{i_1+\dots+i_d} C^F_{\alpha,S}(g_{1,i_1}, \dots, g_{d,i_d}), g_{i,1} = u_i, g_{i,2} = v_i$. In the definition of $\mathrm{V}_{C^F_{\alpha,S}}$ we assume that $u_i \leq v_i$ for all $i \in I$. More generally, we can define $\mathrm{V}_{C^F_{\alpha,S}}(B) = \sum_{\boldsymbol{z} \in \times_{i=1}^d \{u_i,v_i\}} (-1)^{\mathrm{N}_I(\boldsymbol{z},\boldsymbol{u},\boldsymbol{v})} C^F_{\alpha,S}(\boldsymbol{z})$ where $\mathrm{N}_I(\boldsymbol{z},\boldsymbol{u},\boldsymbol{v}) := |\{k \in I | z_k = \min\{u_k, v_k\}\}|$. Hence, there is no need to swap u_i and v_i if $u_i > v_i$ for any i.
Let $B = \times_{i=1}^d [u_i, v_i]$. We define $\psi_S(z_k) := \chi_S(k)(1-z_k) + \chi_{\overline{S}}(k)(z_k)$, where χ is the indicator function:

$$\chi_A(i) := \begin{cases} 1 & \text{if } i \in A, \\ 0 & \text{if } i \notin A. \end{cases}$$

It holds:

$$\begin{aligned}
\mathrm{V}_{C^F_{\alpha,S}}(B) &= \sum_{\boldsymbol{z} \in \times_{i=1}^d \{u_i,v_i\}} (-1)^{\mathrm{N}_I(\boldsymbol{z},\boldsymbol{u},\boldsymbol{v})} \sum_{A \subseteq S} (-1)^{|A|} C_\alpha(\kappa_{S,A}(1,\boldsymbol{z}), \dots, \kappa_{S,A}(d,\boldsymbol{z})) \\
&= \sum_{A \subseteq S} (-1)^{|A|} \sum_{\boldsymbol{z} \in \times_{i=1}^d \{u_i,v_i\}} (-1)^{\mathrm{N}_I(\boldsymbol{z},\boldsymbol{u},\boldsymbol{v})} C_\alpha(\kappa_{S,A}(1,\boldsymbol{z}), \dots, \kappa_{S,A}(d,\boldsymbol{z})) \\
&= (-1)^{|S|} \sum_{\boldsymbol{z} \in \times_{i=1}^d \{u_i,v_i\}} (-1)^{\mathrm{N}_I(\boldsymbol{z},\boldsymbol{u},\boldsymbol{v})} C_\alpha(\kappa_{S,S}(1,\boldsymbol{z}), \dots, \kappa_{S,S}(d,\boldsymbol{z})) \\
&\quad + \underbrace{\sum_{\substack{A \subseteq S \\ A \neq S}} (-1)^{|A|} \sum_{\boldsymbol{z} \in \times_{i=1}^d \{u_i,v_i\}} (-1)^{\mathrm{N}_I(\boldsymbol{z},\boldsymbol{u},\boldsymbol{v})} C_\alpha(\kappa_{S,A}(1,\boldsymbol{z}), \dots, \kappa_{S,A}(d,\boldsymbol{z}))}_{=0, \text{ see } (6)} \\
&= (-1)^{|S|} \sum_{\boldsymbol{z} \in \times_{i=1}^d \{u_i,v_i\}} (-1)^{\mathrm{N}_I(\boldsymbol{z},\boldsymbol{u},\boldsymbol{v})} C_\alpha(\chi_S(1)(1-z_1) + \chi_{\overline{S}}(1)(z_1), \dots, \chi_S(d)(1-z_d) + \chi_{\overline{S}}(d)(z_d)) \\
&\stackrel{(7)}{=} (-1)^{|S|} \sum_{\boldsymbol{z} \in \times_{i=1}^d \{u_i,v_i\}} (-1)^{\mathrm{N}_I(\psi_S(\boldsymbol{z}),\psi_S(\boldsymbol{u}),\psi_S(\boldsymbol{v}))} (-1)^{|S|} C_\alpha(\psi_S(\boldsymbol{z})) \\
&\stackrel{(8)}{=} \sum_{\boldsymbol{z} \in \times_{i=1}^d \{\psi_S(u_i),\psi_S(v_i)\}} (-1)^{\mathrm{N}_I(\boldsymbol{z},\boldsymbol{u},\boldsymbol{v})} C_\alpha(\boldsymbol{z}) = \mathrm{V}_{C^\alpha}(\psi_S(B)).
\end{aligned}$$

(6) For $S = \emptyset$ we have $C^F_{\alpha,S} = C_\alpha$. Therefore, suppose $S \neq \emptyset$. For $A \subseteq S, A \neq S$ there is a $s_A \in S \setminus A$ with $\kappa_{S,A}(s_A, z) = 1$. Hence:

$$\begin{aligned}
&\sum_{\substack{A \subseteq S \\ A \neq S}} (-1)^{|A|} \sum_{\boldsymbol{z} \in \times_{i=1}^d \{u_i,v_i\}} (-1)^{\mathrm{N}_I(\boldsymbol{z},\boldsymbol{u},\boldsymbol{v})} C_\alpha(\kappa_{S,A}(1,\boldsymbol{z}), \dots, \kappa_{S,A}(d,\boldsymbol{z})) \\
&= \sum_{\substack{A \subseteq S \\ A \neq S}} (-1)^{|A|} \left(\sum_{\substack{\boldsymbol{z} \in \times_{i=1}^d \{u_i,v_i\} \\ z_{s_A} = u_{s_A}}} (-1)^{\mathrm{N}_I(\boldsymbol{z},\boldsymbol{u},\boldsymbol{v})} C_\alpha(\dots) + \sum_{\substack{\boldsymbol{z} \in \times_{i=1}^d \{u_i,v_i\} \\ z_{s_A} = v_{s_A}}} (-1)^{\mathrm{N}_I(\boldsymbol{z},\boldsymbol{u},\boldsymbol{v})} C_\alpha(\dots) \right) \\
&= \sum_{\substack{A \subseteq S \\ A \neq S}} (-1)^{|A|} \left(\sum_{\substack{\boldsymbol{z} \in \times_{i=1}^d \{u_i,v_i\} \\ z_{s_A} = u_{s_A}}} \left((-1)^{\mathrm{N}_I(\boldsymbol{z},\boldsymbol{u},\boldsymbol{v})} C_\alpha(\dots) + (-1)^{\mathrm{N}_I(\boldsymbol{z},\boldsymbol{u},\boldsymbol{v})-1} C_\alpha(\dots) \right) \right)
\end{aligned}$$

$$= \sum_{\substack{A \subseteq S \\ A \neq S}} (-1)^{|A|} \sum_{\substack{\boldsymbol{z} \in \times_{i=1}^{d} \{u_i, v_i\} \\ z_{S_A} = u_{S_A}}} 0 = 0.$$

(7) We show $(-1)^{\mathrm{N}_I(\psi_S(\boldsymbol{z}),\psi_S(\boldsymbol{u}),\psi_S(\boldsymbol{v}))} = (-1)^{\mathrm{N}_I(\boldsymbol{z},\boldsymbol{u},\boldsymbol{v})}(-1)^{|S|}$. Let $k := |\{z(i) = u(i) | i \in I \setminus S\}|$ and $l := |\{z(i) = v(i) | i \in S\}|$. Then $\mathrm{N}_I(\boldsymbol{z},\boldsymbol{u},\boldsymbol{v}) = k + |S| - l$ and $\mathrm{N}_I(\psi_S(\boldsymbol{z}),\psi_S(\boldsymbol{u}),\psi_S(\boldsymbol{v})) = k + l$.

$$(-1)^{\mathrm{N}_I(\boldsymbol{z},\boldsymbol{u},\boldsymbol{v})}(-1)^{|S|} = (-1)^{k+2|S|-l} = (-1)^{k-l} = (-1)^{k+l} = (-1)^{\mathrm{N}_I(\psi_S(\boldsymbol{z}),\psi_S(\boldsymbol{u}),\psi_S(\boldsymbol{v}))},$$

where we used that $(-1)^{2|S|} = (-1)^{|S|}(-1)^{|S|} = (-1)^{|S|}/(-1)^{|S|} = 1$ and $(-1)^{-l} = (-1)^{l}$.

(8) $\psi_S : [0,1]^d \to [0,1]^d$ is bijective.

C_α is d-increasing since it is a copula. Thus, $\mathrm{V}_{C^F_{\alpha,S}}(B) = \mathrm{V}_{C^\alpha}(\phi_S(B)) \geq 0$. This completes the proof.

□

References

[1] Comtet L (1974) Advanced Combinatorics: the Art of Finite and Infinite Expansions. Dordrecht: Reidel, 176–177 pp.
